# Portable Cold Atmospheric Plasma Patch‐Mediated Skin Anti‐Inflammatory Therapy

**DOI:** 10.1002/advs.202202800

**Published:** 2022-09-30

**Authors:** Namkyung Kim, Seunghun Lee, Soyoung Lee, Jinjoo Kang, Young‐Ae Choi, Jeongsu Park, Chul‐Kyu Park, Dongwoo Khang, Sang‐Hyun Kim

**Affiliations:** ^1^ Cell & Matrix Research Institute Department of Pharmacology School of Medicine Kyungpook National University Daegu 41944 South Korea; ^2^ Department of Nano‐Bio Convergence Nano Surface Materials Division Korea Institute of Materials Science Changwon 51508 South Korea; ^3^ Immunoregulatory Materials Research Center Korea Research Institute of Bioscience and Biotechnology Jeongeup 56212 South Korea; ^4^ Department of Physiology School of Medicine Gachon University Incheon 21999 South Korea

**Keywords:** calcium channels, cold atmospheric plasma, portable electronic applications, psoriasis, reactive nitrogen species, reactive oxygen species

## Abstract

Although plasma is a promising technology in various fields, its clinical application is restricted by several limitations. A cold atmospheric plasma (CAP) patch is fabricated to help overcome hurdles, especially when treating skin diseases. This patch has surface dielectric barrier discharge, which generates reactive oxygen species (ROS) and reactive nitrogen species (RNS) on a flexible polymer film surface on which the embedded electrode induces a locally strong electric field. The effect of the CAP patch on psoriasis is also evaluated. The distinct characteristics of psoriasis between the lesion and non‐lesion area allow the CAP patch to be suitable for only lesion area for its treatment. The CAP patch induces the opening of calcium channels in keratinocytes, thereby restoring abnormal keratinocyte differentiation and the collapse of the tight junction; thus, alleviating psoriatic symptoms. In addition, the favorable effect is due to the induction of ROS/RNS by the CAP patch, not the electric field generated during plasma generation. The findings indicate that the proposed portable CAP patch can help treat inflammatory skin disorders, especially psoriasis. As this can be used easily as a combination therapy with existing drugs, it may help reduce side effects caused by existing drugs.

## Graphical abstract

The cold atmospheric plasma patch (CAP patch) manufactured for skin inflammation appears to have an alleviating effect by influxing calcium into the lesional area, which is lacking calcium.

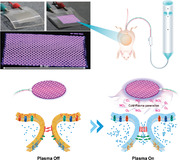

## 1 Introduction

Many basic technologies in medical physics are established through well‐known physics principles. The medical applications of these technologies have improved significantly over the years.^[^ ^1^ ^]^ Among these technologies, psoralen and ultraviolet (PUVA) have helped effectively treat certain skin diseases such as psoriasis and atopic dermatitis. However, long‐term use of PUVA causes a higher risk for skin cancer.^[^ ^2^ ^]^ As there is no perfect cure for skin disease, generally requiring long‐term treatment, there is an urgent need to develop a supplemental treatment with fewer side effects.

Plasma technology has been proved to be promising in various fields such as chemistry, biology, physics, and biotechnological and medical science.^[^ ^3^ ^]^ Recently, the potential for skin treatment via dielectric barrier discharge plasma has also been demonstrated.^[^ ^4^, ^5^ ^]^ Plasma can be generated artificially by heating a gas or applying electromagnetic fields. In this manner, cold atmospheric plasma (CAP), partially ionized gas at atmospheric pressure and room temperature, is capable of generating reactive oxygen species (ROS), reactive nitrogen species (RNS), electric fields, ions, electrons, and ultraviolet (UV) and visible rays.^[^ ^6^, ^7^, ^8^, ^9^, ^10^, ^11^ ^]^ As heat‐generated plasma can adversely affect the skin, low‐temperature atmospheric plasma is required. The atmospheric pressure plasma jet (APPJ) generates plasma by applying a sinusoidal voltage (2–6 kV_pp_) with a frequency of ≈1.0 MHz.^[^ ^12^ ^]^ In addition, kHz frequency APPJ has shown promising therapy results at cancer, wound healing, and antibacterial action.^[^ ^13^, ^14^, ^15^, ^16^, ^17^, ^18^, ^19^, ^20^, ^21^, ^22^ ^]^ These effects might rely on the combined action such as ROS, RNS, free radicals, UV photons, charged particles, and electric fields.^[^ ^23^, ^24^, ^25^, ^26^ ^]^ However, despite having these benefits, it also has limitations such as a narrow range of treatment area, gas supply, and thermal heating. Nowadays, plasma multi jets or plasma arrays have been developed, offering larger surfaces, with very controlled thermal load compatible with tissue treatment but requiring a noble gas supply.^[^ ^27^, ^28^, ^29^, ^30^ ^]^

We fabricated a flexible CAP patch consisting of a portable power unit to overcome these issues. The flexible CAP patch is a polymer film with printed metal electrodes. The CAP patch uses surface dielectric barrier discharge to generate ROS and RNS on flexible polymer film surfaces. The printed metal electrodes induce an electric field of 10–30 kV cm^−1^. A patient will remain safe during the CAP operation despite the strong electric field because the metal electrodes in contact with the skin are electrically grounded while applying a high voltage to the insulated electrode.

We chose psoriasis to assess the clinical application of the CAP patch among all inflammatory skin diseases for two reasons. First, the prevalence of psoriasis among the major inflammatory skin diseases is increasing steadily with no clear treatment. Second, considering the properties of the patch (a narrow range of treatment area), distinguishing between lesion and non‐lesion sites is easy for psoriatic lesions. Psoriasis is an inflammatory skin disease characterized by hyper‐proliferation of keratinocytes, impaired barrier function, and altered calcium gradients in the lesional area.^[^ ^31^, ^32^ ^]^ Calcium in the skin is an important factor for the differentiation of keratinocytes and assembly of tight junctions. In addition, abnormal differentiation of keratinocytes and disruption of tight junctions have been observed due to decreased intracellular calcium ion (Ca^2+^) levels in psoriatic lesions.^[^ ^33^, ^34^, ^35^ ^]^ Psoriatic lesion characterized by lack of Ca^2+^ level is limited only to the lesion area and not seen in the non‐lesion area.^[^ ^36^, ^37^, ^38^ ^]^ Therefore, controlling the Ca^2+^ level in the lesional area is critical for psoriasis management. Treatment via calcium activity during psoriatic progressions could be a critical factor. Given this, we reported the probability of clinical application of the CAP patch for treating psoriasis. We anticipate that the CAP patch could be a candidate as a supplemental treatment approach for inflammatory skin diseases, especially psoriasis.

## 2 Results and Discussion

### 2.1 Development of CAP Patch Device

**Figure 1:**
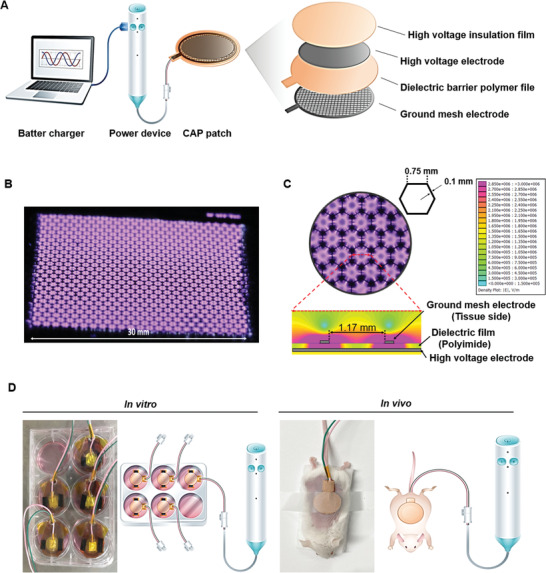
CAP patch devices for in vivo and in vitro tests. A) Portable power unit and circular CAP patch (diameter: 1.5 cm). B) CAP patch with a hexagonal patterned mesh electrode (discharge area: 30 mm × 30 mm). C) Image of surface discharge on CAP patch with a hexagonal pattern and the cross‐sectional view of electric fields at 2 kV input voltage (numerical simulation). D) Setup of in vivo and in vitro experiments.

The CAP patch device used in psoriasis animal testing consists of a CAP patch and a portable power unit (PPU) (**Figure** **1**A). The CAP patch is a combination of high voltage insulating film, dielectric barrier polymer film, high voltage electrode, and a ground mesh electrode (Figure 1A). The details of the CAP patch are described in the section Cap Patch Fabrication of Experimental Section. The CAP patch uses 120 µm – thick polyimide film as a flexible dielectric barrier, which enables the attachment of the patch on curved skin surfaces (Figure 1B). The mesh electrode is fabricated on the film by a screen‐printing method, which makes it easy to expand the area of the CAP patch up to 100 cm^2^. The PPU consists of the main control unit, high voltage power supply, and lithium‐ion battery. The PPU supplies sinewave high voltage (the maximum voltage: 2–2.5 kV). The CAP patch generates surface plasma using ambient air. The air plasma is generated at the edge of the hexagonal electrode. Figure 1C shows the air plasma at hexagonal mesh electrodes on polyimide films. When the PPU supplies 2 kV sinewave voltage, the intensity of electric fields at the edge of the mesh electrode is above 30 kV cm^−1^, enough to induce air plasma on the patch surface. At the maximum voltage of 2 kV, the pink region in the cross‐sectional structure of the patch shows a region where electric field intensity is higher than 30 kV cm^−1^ (Figure 1C). This image is numerically calculated Laplacian electric field by finite‐elements simulation software (https://www.femm.info). Figure 1C does not consider space charge distribution in discharge region.^[^ ^39^, ^40^, ^41^, ^42^ ^]^ To cover the mice skin and well‐plates, we used the circular CAP patch with a diameter of 1.5 cm in the in vivo and in vitro experiments (Figure 1D).

### 2.2 Characteristics of CAP Patch Device

**Figure 2:**
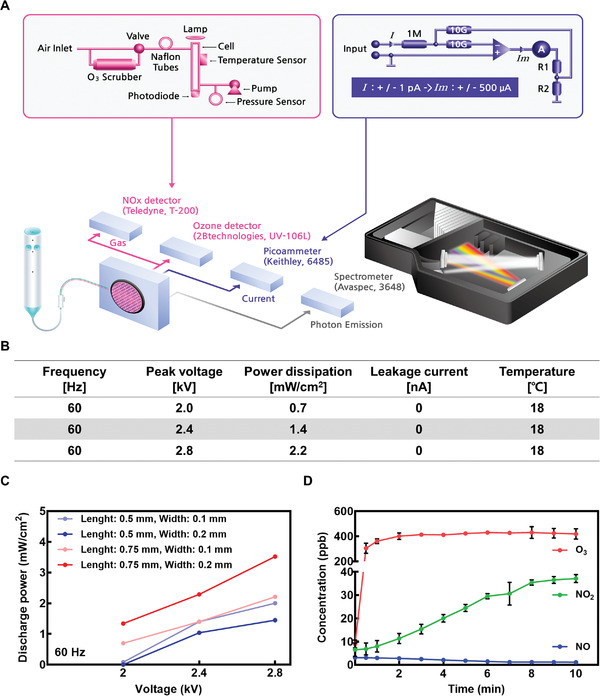
The diagnosis of CAP patch. A) The illustration of the experimental setup of CAP patch diagnostics: gas concentration, leakage current, and optical emission. B) Diagnostic results: power dissipation by plasma, leakage current, and temperature on the CAP patch surface at different peak voltages. C) Power dissipation by plasma as a function of unit line length and width at 60 Hz discharge. D) Ozone, NO, and NO_2_ generation by CAP patch (operation time: 0–10 min).

The characteristics of the CAP patch, such as power dissipation, leakage current, surface temperature, and optical emissions, are measured using a diagnostic system (**Figure** **2**A). The CAP patch is attached to the wall of the diagnostic system. The wall has an electric current probe, an optical lens with a cosine collector, and a gas sampling port. The power dissipation is measured from the PPU signal. A plasma patch supplies ozone to the skin. Therefore, the time‐weighted average (TWA) for working hours per day must comply with the Korea “Exposure Standards for Chemicals and Physical Factors”. TWA refers to the exposure time based on 8 h per day, calculated according to the following formula.

(1) TWA=∑CT/8

where *C* is the concentration of ozone (ppm in volume) and *T* is the exposure time (h). According to the “Guidelines for Approval Review for Plasma‐Generating Medical Devices Used for Skin” recently issued in Korea, the TWA of plasma skin treatment devices is restricted to be less than 0.05 ppm. Table S1, Supporting Information shows the TWA values according to the operating conditions of the CAP patch. The daily usage time was 10 min (1/6 hr), and the ozone concentration according to operating frequency was applied to calculate TWA. Under the 60 Hz frequency condition, TWA satisfies the regulation value of 0.05 ppm. However, it exceeds 0.05 ppm at 1000 Hz. Therefore, the CAP patch was operated at 60 Hz during in vivo and in vitro tests because frequent discharge repetition could induce thermal, optical, and electrical damage. Power dissipation in 60 Hz frequency was in the range of 0.7–2.2 mW cm^2^, where power consumption did not increase the surface temperature from the initial temperature (18 °C) (Figure 2B). The power dissipation was measured by *Q–V* Lissajous diagram method. The optical emission and leakage current were not detectable in the diagnostic system. The hexagonal pattern geometry, such as line width and length were optimized for enhancing plasma generation (Figure 2C). The optimized geometry of mesh electrode had a line length of 0.75 mm and a line width of 0.2 mm. This pattern was applied to in vivo and in vitro tests. The concentration of ROS and RNS supplied by CAP patch was measured by ozone and NO*_x_* detectors. In 2.8 kV discharge, O_3_ concentration was rapidly increased to 400 ppb and further saturated. The concentration of NO was under 5 ppb. The concentration of NO was similar to that of initial gas concentration. The concentration of NO_2_ was increased continuously to 38 ppb, which is under the regulation limit of 1‐hour exposure (100 ppb) (Figure 2D). In practical use, the ROS and RNS species are difficult to breathe through the respiratory tract due to the distance from the nose.

### 2.3 CAP Patch Induces an Influx of Ca^2+^ Through Ca^2+^ Channels Opening

Calcium ions are important to regulate many skin functions such as keratinocyte differentiation and skin barrier formation.^[^ ^43^ ^]^ In psoriatic keratinocytes, abnormal differentiation and tight junction instability are observed due to insufficient calcium influx.^[^ ^34^, ^44^ ^]^ Considering the importance of calcium in keratinocytes, it was confirmed whether the CAP patch could induce calcium influx into cells.

**Figure 3:**
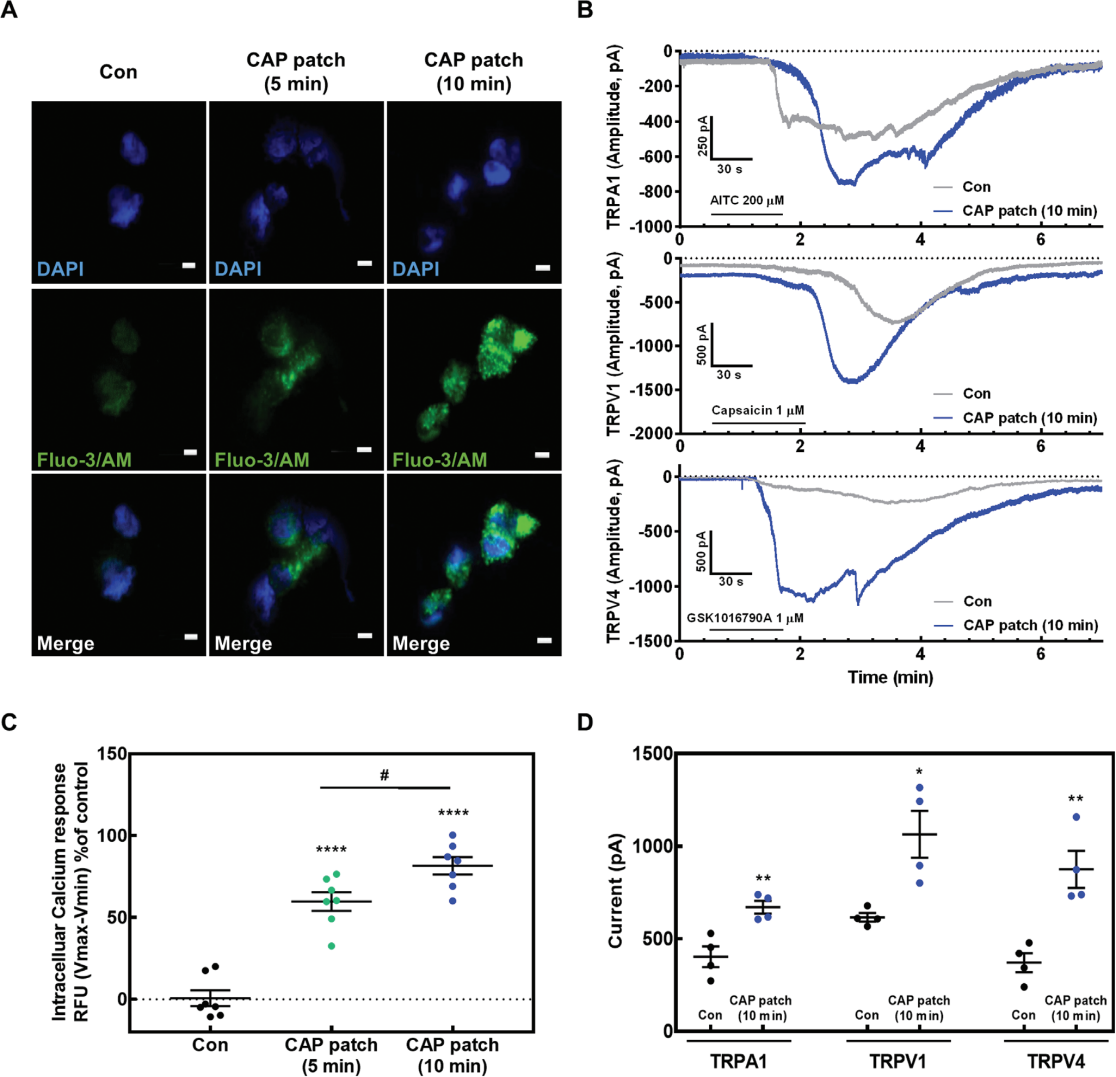
CAP patch induced the influx of Ca^2+^ via opening calcium channels. A) Calcium image of intracellular calcium influx and C) intracellular calcium influx of keratinocytes by CAP patch. B) Representative current trace elicited by 200 µм AITC (TRPV1 agonist), 1 µм capsaicin (TRPV1 agonist), and 1 µм GSK 1016790A (TRPV4 agonist) in keratinocytes before (CON; upper) and after treatment with CAP patch for 10 min (bottom). D) The current amplitude values comparison of mean inward currents. The data are presented as mean ± SEM (*n* = 4). * *p* < 0.05 compared with CON group. # *p* < 0.05 compared with CAP patch (5 min).

To assess the calcium influx, we performed fluorescence analysis using a calcium indicator (Fluo‐3/AM). Confocal microscopy and fluorometry revealed that intracellular calcium level increased after CAP patch treatment in keratinocytes (**Figure** **3**A). In addition, patch clamps were performed to determine which calcium channels led to calcium influx. In the calcium channels, various transient receptor potential (TRP) channels participate in the formation and maintenance of skin barrier, as well as cutaneous immunological and inflammatory processes; thereby, establishing skin homeostasis, along with contributing to many types of skin disorders.^[^ ^44^, ^45^ ^]^ TRP channels are not only expressed in sensory nerve endings but also in many non‐neuronal cell populations including keratinocytes and skin‐resident immune cells.^[^ ^19^ ^]^ Especially, TRP subfamily V member 1, TRP subfamily A member 1, and TRPV4 have been shown to control psoriasis.^[^ ^46^, ^47^, ^48^, ^49^ ^]^ Thus, we used whole‐cell voltage‐clamp analysis to examine the electrophysiological responses to the following agonists: AITC (200 µм, for TRPA1), Capsaicin (1 µм, for TRPV1), and GSK1016790A (1 µм, for TRPV4), in similar sized control and CAP patch treated keratinocytes. The current amplitude induced by respective agonists for TRPA1, TRPV1, and TRPV4 was significantly increased without specific change of cell morphology by CAP patch treatment for 10 min (Figure S1, Supporting Information), compared to the control in TRPA1, TRPV1, and TRPV4. This indicates that the CAP patch induces the opening of calcium channels (TRPA1, TRPV1, and TRPV4), leading to an increase in calcium levels in keratinocytes (Figure 3B,C).

### 2.4 CAP Patch Suppresses the Inflammatory Response and Disruption of Tight Junctions in Activated Keratinocytes

**Figure 4:**
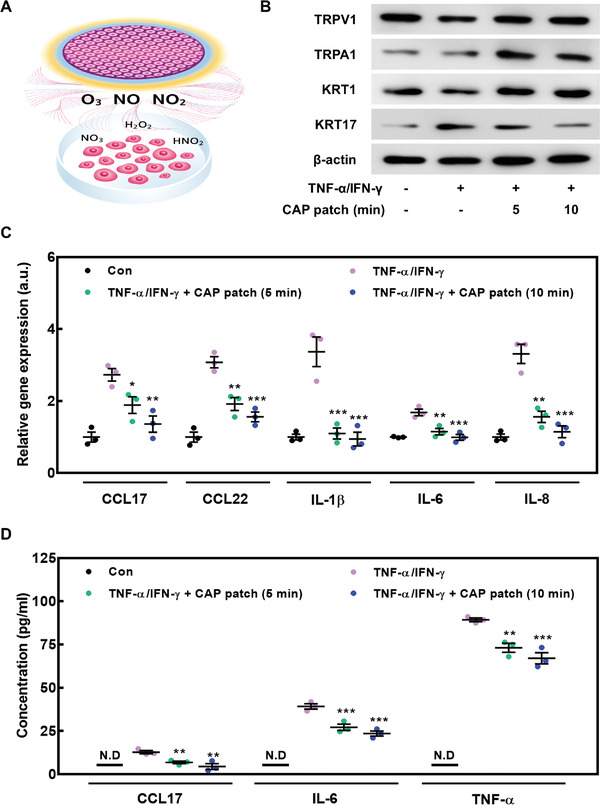
CAP patch decreased psoriasis‐associated gene expression and protein level as well as induced the normal differentiation of keratinocytes. To assess beneficial effects of CAP patch in keratinocytes, cells were activated with TNF‐*α* and IFN‐*γ* (10 ng mL^−1^) with CAP patch (5 or 10 min) for 6 h (for qPCR) or 24 h (for ELISA and Western blot). A) Schematic image of in vitro experiment. B) TRPV1, TRPA1, KRT17, KRT1, and *β*‐actin were detected using Western blot. *β*‐actin was used as a loading control. C) The gene expression of cytokines and chemokines in cells was measured using qPCR and normalized to GAPDH. D) The cells were activated with TNF‐*α* and IFN‐*γ* (10 ng mL^−1^) and then treated with the CAP patch (5 or 10 min) for 24 h. After 24 h, supernatants were obtained. Subsequently, the protein levels in the supernatant were measured by ELISA. The data are presented as mean ± SEM (n = 3). * *p* < 0.05 compared with the TNF‐*α*/IFN‐*γ*‐stimulated group only.

To assess the anti‐inflammatory role of CAP patch in keratinocytes, we performed in vitro experiments as shown in **Figure** **4**A. We made a hole on the lid of the 6‐well plate and inserted the CAP patch inside to prevent the plasma from flowing out of the plate. We investigated whether the CAP patch inhibits the disruption of keratinocytes induced by TNF‐*α*/IFN‐*γ*, the two top inflammatory mediators increased in serum of psoriasis patients and in vitro psoriasis model.^[^ ^50^ ^]^ We co‐treated TNF‐*α*/IFN‐*γ* in keratinocytes and then treated CAP patch for 5 or 10 min. TNF‐*α*/IFN‐*γ* decreased keratin (KRT)1 and increased KRT17 in keratinocytes, which induced a keratin pattern similar to that of psoriasis.^[^ ^51^ ^]^ However, CAP patch restored KRT1 associated with normal differentiation and inhibited KRT17 associated with aberrant differentiation. We also confirmed that TRPV1 and TRPA1 were increased by CAP patch (Figure 4B; Figure S2, Supporting Information). These results imply that CAP patch blocks abnormal keratin differentiation by increasing calcium influx. In addition, while TNF‐*α*/IFN‐*γ* increased the gene expression of inflammatory cytokines (IL‐1*β*, IL‐6 and IL‐8) and chemokines (CCL17 and CCL22) in keratinocytes, expression was decreased by CAP patch (Figure 4C). The secretion of cytokines (TNF‐*α* and IL‐6) and chemokine (CCL17) by the TNF‐*α*/IFN‐*γ* stimulation was also reduced by the CAP patch (Figure 4D). These results suggest that CAP patch alleviated psoriasis by reducing its stimulated factors. Collectively, this evidence suggests that the fabricated CAP patch can help relieve systemic and local inflammatory reaction in the lesion area of psoriasis, further supporting its usefulness to treat inflammatory skin diseases.

**Figure 5:**
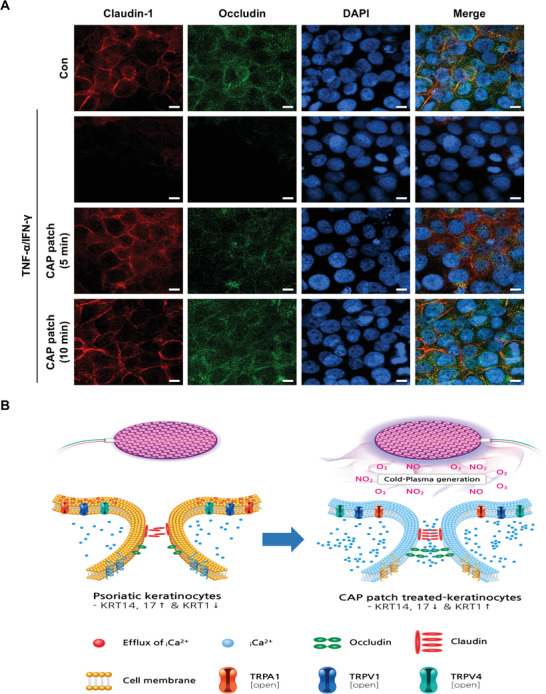
CAP patch inhibited the disruption of tight junctions of keratinocytes. A) At 630× magnification, fluorescence microscopic images of immune‐stained tight junctions (claudin‐1, occludin) in HaCaT cells treated with TNF‐*α*/IFN‐*γ* (20 ng mL^−1^) or CAP patch (5 or 10 min) for 18 h at 37 °C. B) The illustration figure summarizes the results of Figures 3, 4, and 5.

Tight junctions form continuous intercellular barrier between the epithelial cells, which is required to separate tissue spaces and regulate selective movement of solutes across the epithelium.^[^ ^52^ ^]^ Lack of calcium in keratinocytes alters the tight junction activity, particularly of occludin and claudin.^[^ ^43^, ^53^ ^]^ We found that the tight junctions are disrupted by TNF‐*α*/IFN‐*γ* in keratinocytes. However, the CAP patch appeared to inhibit those disruptions, as noted under the confocal microscopic observation (**Figure** **5**A).

Combining the results up to Figures 3, 4, 5, the findings suggest that the CAP patch opened calcium channels and induced calcium influx in keratinocytes; thereby, inhibiting abnormal differentiation, alleviating inflammation‐related cytokines, and inhibiting the disruption of tight junctions (Figure 5B).

### 2.5 CAP Patch Alleviates Lesions With Psoriatic Characteristics

Psoriasis has a variety of common symptoms, including scaling skin, redness and inflammatory keratosis, and abnormal histological changes, including epidermal/dermal thickness within a psoriatic lesion.^[^ ^54^ ^]^ To assess the effect of the CAP patch on psoriasis‐like skin inflammation, we applied imiquimod (IMQ) cream on mice skin for 7 consecutive days. Subsequently, a CAP patch was placed on the mice skin for 5 or 10 min. In our preliminary study, no positive effect was observed when the CAP patch was treated for less than 3 min (data not shown). The treatment using IMQ or CAP patch on the back skin of the mouse was performed constantly at the same time and same skin lesion for 7 consecutive days.

**Figure 6:**
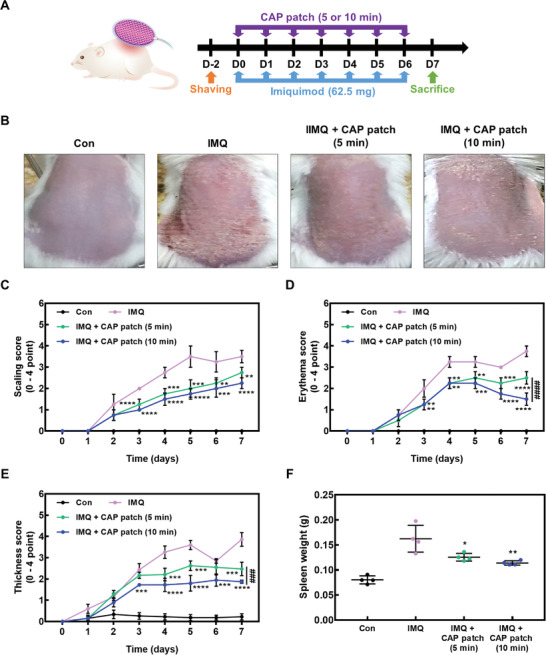
CAP patch alleviated the psoriatic characteristics. A) The experimental scheme of CAP patch effect in IMQ‐induced model. B) Phenotypic observations of dorsal skin. C–E) Scaling, erythema, and thickness were measured by the PASI score method from 0 to 4 for 7 consecutive days. F) Spleen weight of mice. The data are presented as mean ± SEM (*n* = 4). * *p* < 0.05 compared with the IMQ group only. # *p* < 0.05 compared with IMQ plus CAP patch (5 min).

**Figure 7:**
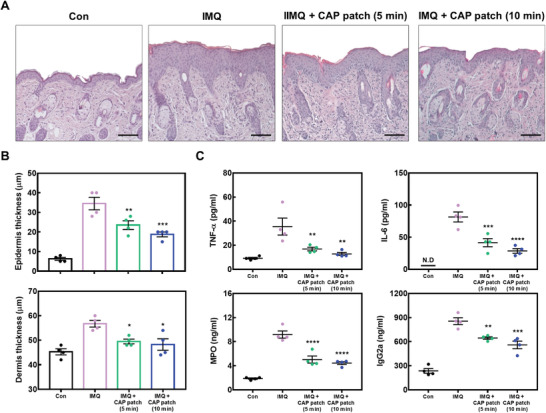
CAP patch decreased the inflammatory mediators in psoriasis. A) Mice skin tissues were stained with H&E to investigate histological observation and for the determination of epidermal and dermal thickness. B) Epidermal and dermal thickness. At 200× magnification, the thickness of the epidermis and dermis was assessed with the stage micrometer. C) IL‐6, TNF‐*α*, IgG2a, and MPO were measured through sandwich ELISA. Whole blood was collected through the abdominal vena cava. Serums from whole blood were separated using centrifugation. * *p* < 0.05 compared with the IMQ group only.

**Figure 8:**
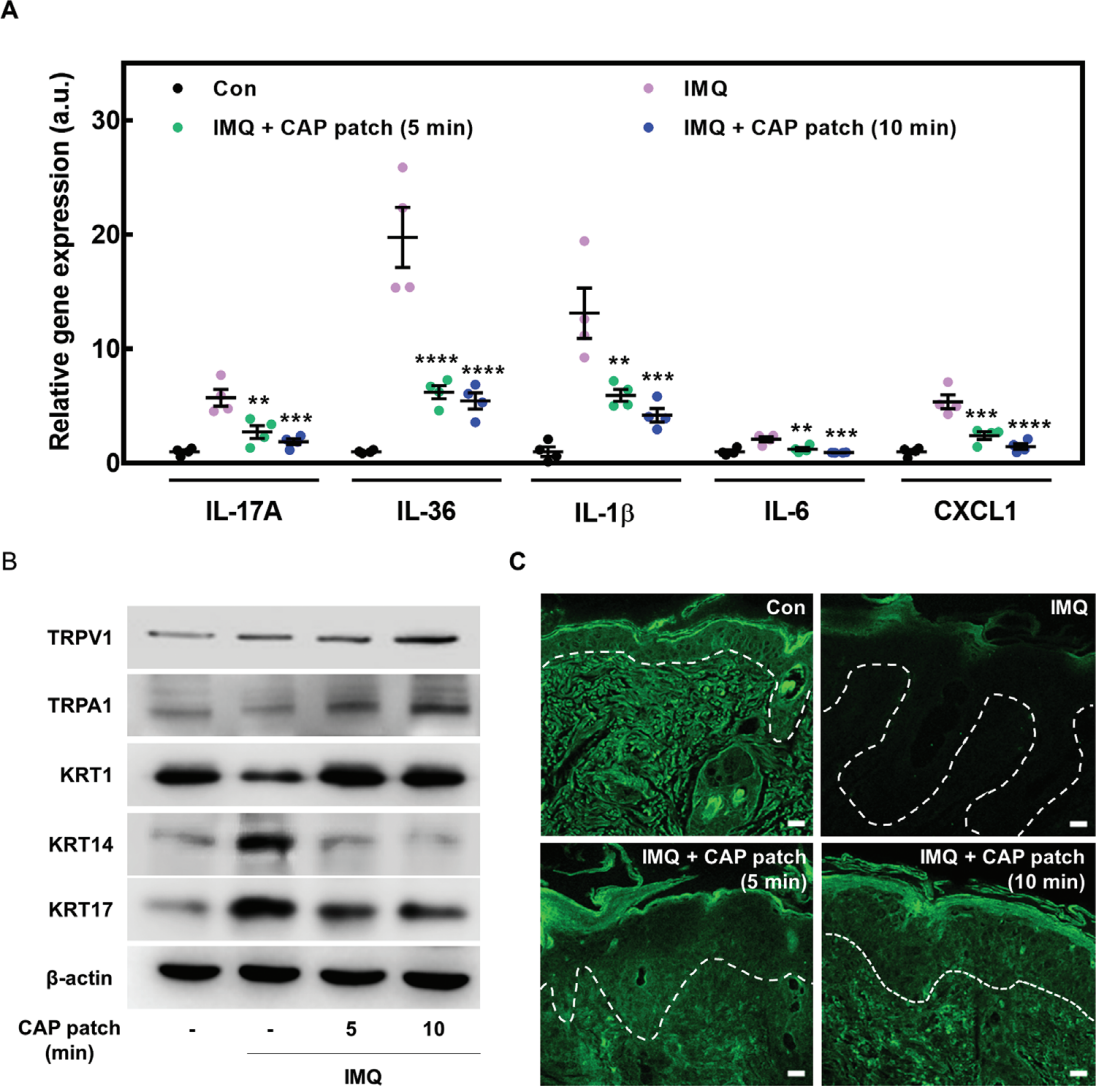
CAP patch interacts with calcium to inhibit the inflammation in lesions. A) mRNA expression of cytokines in mice skin. Mice skin was isolated and total RNA was extracted. The mRNA expression was measured using qPCR and normalized to GAPDH. B) Keratins, TRPV1 and TRPA1 were detected by Western blot in IMQ‐induced skin. C) Calcium gradient image in mice skin. The data are presented as mean ± SEM (*n* = 4). * *p* < 0.05 compared with the IMQ group only.

Experiments were performed according to the scheme shown in **Figure** **6**A. Continual application of IMQ, which is a toll‐like receptor 7/8 agonist, can induce and accelerate psoriasis symptoms.^[^ ^55^, ^56^ ^]^ In addition, infiltrated immune cells in the epidermis and resident keratinocytes continuously interact through the production of various cytokines and chemokines, which aggravates skin inflammation in terms of the immunological aspect of psoriasis.^[^ ^57^ ^]^ Repeated application of IMQ dramatically increases psoriatic conditions. Psoriatic area and severity index (PASI) scoring has been commonly used to assess the severity of psoriasis, including scaling, erythema, and thickness.^[^ ^58^ ^]^ In addition, psoriasis patients develop splenomegaly due to the expression of the immune response to the state of chronic inflammation.^[^ ^59^ ^]^ However, the CAP patch alleviated the IMQ‐induced psoriasis‐like skin inflammation such as scaling, thickness, and spleen weight (Figure 6B–F). In addition, IMQ‐induced skin showed dramatically increased epidermis and dermis thickness compared to the control group, while the CAP patch decreased epidermis and dermis thickness (**Figure** **7**A,B). Furthermore, the CAP patch decreased the psoriasis‐related immunoglobulin (Ig)G2a and cytokines such as IL‐6, TNF‐*α*, and myeloperoxidase (MPO) (Figure 7C). Subsequently, CAP patch attached on lesions reduced the gene expression of inflammation‐related cytokines/chemokines such as IL‐17A, IL‐1*β*, IL‐36, IL‐6, and CXCL1, which was increased by IMQ application (**Figure** **8**A).

The expression levels of KRT5 and KRT14 in the basal cell layer are also altered in the psoriatic epidermis.^[^ ^60^, ^61^ ^]^ In addition, KRT17 is closely related to the immune system and plays a critical role in the pathogenesis of psoriasis.^[^ ^62^ ^]^ These phenomena are caused by decreased calcium levels in the keratinocytes, suggesting that calcium concentration determines keratin differentiation.^[^ ^61^, ^63^ ^]^ Therefore, we confirmed the change of KRTs in IMQ‐induced psoriatic skin. IMQ‐induced skin increased keratin levels of KRT14 and KRT17 associated with abnormal differentiation, while decreasing KRT1. In contrast, the CAP patch restored the KRT1 associated with normal differentiation as well as reduced the KRT14 and KRT17 and enhanced the activation of calcium channel molecules as TRPV1 and TRPA1 (Figure 8B).

To verify whether the CAP patch recovers the calcium gradient in the skin tissue of the psoriatic lesion site, we performed calcium gradient staining to observe its influx by confocal microscopy. We anticipated that calcium would have a good effect on psoriatic lesions because intracellular calcium levels were increased in keratinocytes. As expected, it was confirmed that the reduced calcium gradient was dramatically recovered by the CAP patch (Figure 8C). These results hint at the recovery of psoriatic lesional skin by calcium influx into the skin cells, suggesting that the function of keratinocytes will also depend on the calcium influx into the keratinocytes.

To further confirm the effect of the CAP patch in psoriasis, another well‐known psoriasis model, that is, a topical 12‐O‐tetradecanoylphorbol‐13‐acetate‐induced mouse model, was used to confirm the therapeutic effect of the CAP patch on psoriasis.^[^ ^64^, ^65^ ^]^ We confirmed that the CAP patch also has a relieving effect on the above model without toxicity (Figure S3A–E, Supporting Information). Considered together, our results imply that a topically applied CAP patch relieves the inflammatory response in the lesion area, suggesting that a fabricated CAP patch can be safely and effectively used to treat chronic inflammatory skin diseases, especially psoriasis.

### 2.6 The Effect of the CAP Patch is the Effect of the Generated Plasma, Not of the Generated Electric Field

To determine whether these favorable effects are due to plasma or an electric field generated during plasma generation, we fabricated a plasma‐free patch that does not generate plasma. It is commonly known that an electric field is generated during plasma generation. This electric field interacts with biological substances, which affects the depolarization of nerves and muscles and induces deep tissue heat. In addition, it has been proven to have anti‐cancer effects.^[^ ^66^ ^]^ Therefore, it is worth distinguishing the effectiveness of CAP patch coming from generated plasma and/or generated electric field. For this test, we developed a CAP‐free patch. The electric field intensity distribution in the cross‐section of the CAP patch was calculated using Maxwell's equation. A schematic diagram of the electric field and plasma at the CAP patch surface according to Maxwell's equation is shown in Figure S5, Supporting Information. The plasma is generated through an electric discharge in the air under the condition such that an electric field of 30 kV cm^−1^ or more is generated on the patch surface of the CAP patch. Interestingly, the CAP patch reduced psoriatic characteristics such as epidermal hyperplasia, parakeratosis, scaling, edema, thickness, and erythema. However, no such phenomenon was seen in the electric field patch (Figure S5B–G, Supporting Information).

The tissue stimulation by electric field could be different at CAP patch, DBD, and APPJ. The CAP patch generates air discharge on the patch surface. The air discharge generates gas phase RONS species, which diffuse to the tissue surface through a fabric tape. The thickness of fabric tape makes a gap of 1–1.5 mm between the CAP patch and tissue surface. The electric field on CAP patch surface is attenuated below 1.5 kV cm^−1^ (Figure S4, Supporting Information). In DBD, the electric field in the tip of the filament is of the order of 150–200 kV cm^−1^. When the filament strikes a surface, electric fields being many hundreds of kV cm^−1^, last for a few ns.^[^ ^67^ ^]^

In He/O_2_ APPJ, the electric fields at cell membrane, cytoplasm, and nucleus could be 8–25 kV cm^−1^.^[^ ^68^ ^]^ The electric field at APPJ could be sufficient to induce either electroporation or stimulating voltage‐gated channels in the membranes. The representative APPJ device, kINPen med (Neoplas med),^[^ ^6^ ^]^ has showed that CAP‐derived radicals penetrate human tissue sample,^[^ ^69^ ^]^ and immediate changes of specific molecular tissue components.^[^ ^70^ ^]^

In conclusion, the electric field patch did not show any beneficial effect on IMQ‐induced psoriasis‐associated symptoms and immunological aspects. These results suggest that the generated plasma, not the generated electric field, alleviates the psoriasis symptoms.

### 2.7 The Safety of CAP Patch from the Immunological and Physiological Aspects

**Figure 9:**
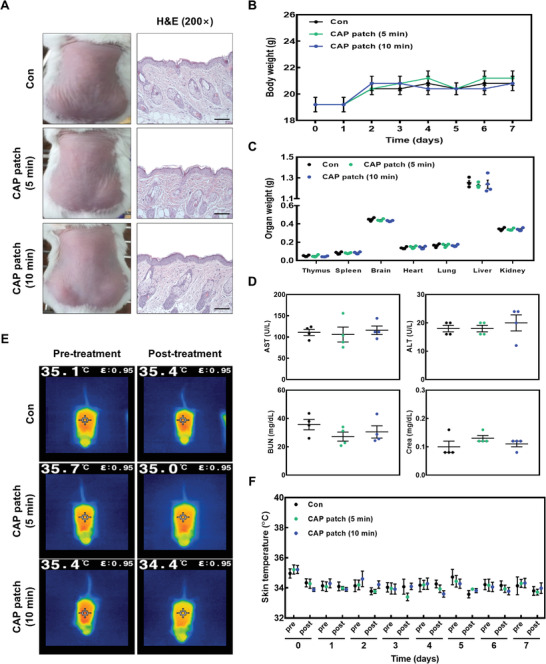
The CAP patch had no effect on serological or immunological aspects in normal condition. A) Representative figure and histological analysis after CAP patch treatment in normal skin. B,C) The body and organs weight after CAP patch treatment for consequent 7 consecutive days. D) After sacrificing, blood was obtained from mice. Biochemical parameters (AST, ALT, BUN, and CREA) of mice treated with CAP patch. E,F) The skin temperature before and after CAP patch treatment.

Biomedical applications of plasma require its efficacy for specific purposes and safety. Indeed, the safety of plasma technology has been proven in various studies.^[^ ^71^, ^72^ ^]^ However, due to the following two reasons, safety verification of CAP patch is essential: 1) CAP patch is used directly on the skin and 2) ROS produced by plasma may cause physiological and pathological skin problems, as reported.^[^ ^73^ ^]^ Hence, we examined how the fabricated CAP patch affects the normal skin for 7 consecutive days. We first analyzed histological observation and local skin temperature as the skin temperature should not exceed 40 °C (hyperthermia) in normal skin.^[^ ^74^ ^]^ In histological analysis, the CAP patch did not exhibit abnormality, including scar, hyperplasia, burn, and edema in mice (**Figure** **9**A). In addition, the CAP patch did not change the body weight and organ weights of mice (Figure 9B,C).

Biochemistry results such as aspartate aminotransferase (AST), alanine aminotransferase (ALT), blood urea nitrogen (BUN), and creatinine (CREA) were not altered by CAP patch treatment (Figure 9D). These results indicate that the fabricated CAP patch is non‐toxic and non‐hazardous, at least in our experimental system. In addition, the CAP patch did not affect the skin temperature during the experimental period in local skin temperature (Figure 9E,F).

To investigate the changes in the local function of the calcium channels by the CAP patch in normal skin, we performed Western blotting with TRPV1 and TRPA1 in mouse tissue. The CAP patch was found to have no effect on the calcium channel (Figure S6, Supporting Information). These results suggest that when the CAP patch is applied to the lesion, it affects the calcium channel. However, it does not affect the calcium channels in normal skin. Subsequently, to verify the unexpected immunotoxicity, we measured the T cell population in the immune organs (spleen and thymus) using flow cytometry. As shown in Figure S7, Supporting Information, the CAP patch did not alter CD4^+^ and CD8^+^ cell populations in the spleen and thymus, respectively. Considered together, these results suggest that the fabricated CAP patch is free from toxicity and safety issues from immunological aspects.

In general, ROS/RNS generated endogenously or in response to environmental stress has long been implicated in tissue injury in the context of a variety of disease states. ROS/RNS causes cell death by nonphysiological (necrotic) or regulated pathways (apoptotic).^[^ ^73^, ^75^ ^]^ Therefore, we assessed changes in the cells upon application of the CAP patch in normal keratinocytes. We found that the CAP patch did not induce cell death in keratinocytes (Figure S8, Supporting Information). Collectively, the CAP patch has no specific side effect definite in terms of physiology and immunology and does not induce cell death either.

Despite various studies on psoriasis, there is currently no cure. Instead, there are various therapeutic options for the treatment of psoriasis, such as topical agents (corticosteroids, vitamin D analogs, retinoids, calcineurin inhibitors, and coal tar), immunosuppressive agents (Methotrexate and Cyclosporine), and biologic agents (Etanercept, Adalimumab, Infliximab, and Ustekinumab).^[^ ^76^, ^77^, ^78^ ^]^ However, the proven existing treatments are applied to both the lesional and non‐lesional areas; therefore, resulting in adverse effects on the latter. Here, we aimed to treat the lesion area exclusively. In our study, we evaluated the possibility of adjuvant treatment for psoriasis by producing a CAP patch in a round shape that can enable its application only on the psoriatic lesions. Consequently, it was confirmed that psoriasis was alleviated by recovering the abnormal keratin and disrupting tight junctions in the psoriatic lesion. Overall, our study demonstrated the possibility of clinical application of the CAP patch as a supplemental treatment for psoriasis. Accordingly, we anticipate that combining existing treatment and the CAP patch will be a promising approach for the treatment of chronic inflammatory skin disease, improving the effectiveness of the treatment.

## 3 Conclusion

In summary, our study started by addressing the unmet need for medical devices to treat skin disease in medicine and discussing the limitations of plasma technology. The CAP patch was analyzed as a new treatment option for various skin diseases, especially psoriasis, by evenly delivering ROS/RNS generated by plasma to the skin without specific toxicity. We showed that the CAP patch dramatically alleviated the psoriatic symptoms by introducing calcium into the lesion area. In addition, it was also confirmed that there were no side effects in terms of physical and immunological aspects when applied directly to the skin. We believe that this CAP patch could be a promising candidate to meet the needs of patients, and with added advantages of portability, safety, and convenience, it can improve user satisfaction. Moreover, as the CAP patch can be applied only to the lesion site, it is expected that the concentration of existing drugs can be lowered, as it can be used easily in combination with existing drugs. The combination of CAP patch and existing drugs will effectively benefit both the patients and the clinicians by suggesting another treatment option for skin diseases.

Although clinical trials in humans must be carried out to conclude the results, this study presented a novel approach to treating target lesions by applying the CAP patch in animal experiments. Therefore, we highlight that CAP patches could potentially advance current psoriasis treatment research and inspire novel treatment options for a wide range of skin conditions.

## 4 Experimental Section

### CAP Patch Fabrication

CAP patch consists of a high voltage electrode, dielectric barrier polymer film, and ground mesh electrode. A high voltage electrode is an aluminum adhesive film. The dielectric barrier film was a commercial polyimide sheet (SKC, Seoul, Republic of Korea) with a thickness of 120 µm. A ground mesh electrode was Ag nanoparticle ink printed by silkscreen printing. The thickness, line width, and mesh size of the ground hexagonal mesh electrode were 3 µm, 0.1 mm, and 0.75 mm, respectively. CAP patch was fabricated by the following steps: 1) cleaning polyimide films with a rubber dust remover, 2) silk screen printing of ground mesh electrode on the polyimide film, 3) attaching high voltage electrode, 4) connecting an electrical cable to both electrodes, 5) insulating high voltage electrode by polyimide tape, and 6) covering the ground mesh electrode by a fabric tape for finishing.

### CAP Patch Diagnostics

The characteristics of the CAP patch, such as ROS and RNS concentration, leakage current, optical emission, and thermal heating, were measured using the diagnostic system (Figure 2A). The chamber volume was 220 mm × 225 mm × 70 mm, and the CAP patch was attached to a wall on which the gas sampling port, current collector, and cosine collector were attached. The chamber wall was insulated by a dielectric film (polyimide). In this work, tissue sample was not covering the wall surface of diagnostic chamber. The effect of target surface has been described in previous works.^[^ ^79^, ^80^, ^81^, ^82^, ^83^ ^]^ Ozone concentration was measured by an ozone detector (2B technologies, UV‐106L, Boulder, CO, USA) using the UV absorption method. Nitric oxide (NO) and nitrogen dioxide (NO_2_) were measured by the NO*_x_* detector (Teledyne, T‐200, San Diego, CA, USA) using UV and ozone methods. The flow rate for gas sampling was minimized to 0.1 ppm to create conditions such that the CAP patch was attached to a skin surface without any flow. A leakage current was collected by a copper electrode connected to a picoammeter (Keithley, 6485, Cleveland, OH, USA). Optical emission was collected by a cosine collector and transmitted to a spectrometer (Avaspec, 3648, Apeldoorn, Netherlands) by UV–vis optical fiber. The surface temperature of the CAP patch during the operation was measured using an infrared image camera (Fluke, TiS40, Everett, WA, USA) facing the surface of the ground mesh electrode.

### Cell Culture

The human keratinocyte cell line, HaCaT (American Type Culture Collection, Manassas, VA, USA) was maintained in Dulbecco's modified Eagle medium (DMEM, Gibco, Grand Island, NY, USA), supplemented with 10% fetal bovine serum (FBS; Gibco) and antibiotics (100 U mL^−1^ Penicillin G and 100 µg mL^−1^ Streptomycin; Gibco) at 37 °C in 5% CO_2_. Unless stated otherwise, all reagents were purchased from Sigma–Aldrich Co. LLC (St. Louis, MO, USA).

### Animals and Ethics Statements

Eight‐week‐old BALB/c mice (female) were purchased from Dae‐Han Experimental Animal Center (Daejeon, Republic of Korea). The mice were maintained in a laminar‐air‐flow room at a temperature of 22 °C ± 2 °C, relative humidity of 55% ± 5%, and a 12 h light/dark cycle for the study period. All the experimental procedures were in accordance with the guidelines established by the Public Health Service Policy on the Humane Care and Use of Laboratory Animals and were approved by the Institutional Animal Care and Use Committee of Kyungpook National University (KNU‐2020‐0080).

### Induction of Psoriasis‐Like Skin Inflammation in Mice

Psoriasis‐like skin inflammation in mice skin was induced as described previously.^[^ ^55^, ^56^ ^]^ Briefly, mice were shaved using shaving cream and then applied a topical treatment daily of 62.5 mg of IMQ cream (5% Aldara; DONG‐A Pharmaceuticals, Seoul, Republic of Korea) on the dorsal skin. Mice were randomly divided into four groups: 1) control, 2) IMQ, 3) IMQ + CAP patch (5 min), and 4) IMQ + CAP patch (10 min). IMQ was applied topically on mice skin. After 3 h, mice were injected intraperitoneally for anesthesia (Ketamine: Rompun: PBS = 2: 1: 7). Subsequently, a CAP patch was applied to the mice skin for 5 or 10 min. Every 24 h, mice skin was investigated for PASI, skin thickness, and body weight. PASI was measured by scaling, erythema, and thickness, which was scored independently on a five‐point scale from 0–4, as follows: 0, none; 1, mild; 2, moderate; 3, marked; 4, very marked. Skin thickness was measured by a dial thickness gauge (Mitutoyo Co., Tokyo, Japan). The body weight of mice were weighed using an HKC65050 electronic balance (Cas, Incheon, South Korea) at 24 h.

All mice were euthanized using CO_2_ gas. Subsequently, samples such as dorsal skin, organs, and whole blood were collected. Dorsal skin was assessed by histological analysis, qPCR, and Western blot. Serum was obtained from whole blood through centrifuging (400 × *g* for 15 min at 4 °C).

### Enzyme‐Linked Immunosorbent Assay (ELISA)

Serum levels of IgG2a, IL‐6, and MPO were measured using specific ELISA kits according to manufacturers’ protocols. The absorbance was determined at 450 nm with a spectrophotometer (VersaMax; Molecular Devices, LLC, San Jose, CA, USA). The calculation and analysis of data were obtained by using SoftMax Pro 6 software.

### RNA Isolation and Quantitative Real‐Time PCR (qPCR)

HaCaT (1 × 10^6^ cells per well) was seeded in a six‐well plate and pretreated with CAP patch for 5 or 10 min, and further treated with TNF‐*α* (10 ng mL^−1^) and IFN‐*γ* (10 ng mL^−1^) for 6 h at 37 °C in a 5% CO_2_ incubator. After sacrificing, the back skin of the experimental mice was isolated. RNA samples were isolated from back skin tissues and HaCaT cells by using the RNAiso Plus kit (Takara Bio, Shiga, Japan) according to the manufacturer's respective protocols. The skin tissues and cells were prepared at the end of the experimental period. For the synthesis of cDNA, the RevertAid RT kit (Thermo Fisher Scientific, Waltham, MA, USA) was used according to the manufacturer's protocol. The qPCR step was performed by using QGBlue PCR 2X Master Mix (Cellsafe, Yongin, Republic of Korea). Table S2, Supporting Information, shows the specific primers and conditions used for experiments. The mRNA expression was normalized to the respective glyceraldehyde 3‐phosphate dehydrogenase (GAPDH) expression and was analyzed using StepOnePlus (Thermo Fisher Scientific) supplied by the manufacturer.

### Histological Examination

Skin tissues of mice were fixed in 10% formaldehyde and embedded in paraffin. Subsequently, 6 µm sections were stained using hematoxylin and eosin (H&E) according to standard procedures. The epidermis and dermis of tissue were observed and analyzed using ZEISS HBO 100 microscope (Carl Zeiss, Oberkochen, Germany) at 200× magnification. The epidermal and dermal thickness of H&E‐stained skin tissues were measured using a stage micrometer 10:100 microscopic lens (Carl Zeiss).

### Western Blot

HaCaT (1 × 10^6^ cells per well) was seeded in a six‐well plate. Subsequently, cells were treated with a CAP patch for 5 or 10 min and then treated with TNF‐*α* (10 ng mL^−1^) and IFN‐*γ* (10 ng mL^−1^) for 24 h at 37 °C in a 5% CO_2_ incubator. After stimulation, the cells were washed two times with cold PBS (with 100 nm Na_3_VO_4_), and cell lysates were obtained in 100 µL of RIPA (Biosesang, Seongnam, Republic of Korea) containing a protease/phosphatase inhibitor cocktail (Roche, Mannheim, Germany). The lysates were centrifuged at 1200 × *g* for 15 min at 4 °C, and the supernatant was collected. After sacrificing mice, back skin was isolated. Mice tissues were gathered in 300 µL of RIPA containing a protease/phosphatase inhibitor cocktail. The lysates were centrifuged at 1200 × *g* for 15 min at 4 °C, and the supernatant was collected. The whole‐cell lysate protein quantification assay was performed using the Bradford Protein assay kit (Bio‐Rad Laboratories, Hercules, CA, USA) and loaded onto 10% sodium dodecyl sulfate‐polyacrylamide gel electrophoresis, and then transferred onto nitrocellulose membranes (Pall Life science, Port Washington, NY, USA). Membranes were then observed with Ponceau S stain for equivalent loading. The details of the antibodies used for Western blot are summarized in Table S3, Supporting Information. Immunodetection was performed using SuperSignal West Pico Chemiluminescent Substrate (Thermo Fisher Scientific) by G: BOX Chemi XRQ (Syngene, Cambridge, UK).

### Intracellular Calcium Assay and Calcium Image in Keratinocytes

Fluo‐3/AM (Invitrogen, Carlsbad, CA, USA), a fluorescent indicator, was used to measure intracellular calcium levels and calcium image. To assess intracellular calcium levels, cells (1 × 10^4^ cells per well) were seeded in a black 96‐well plate (Greiner Bio‐One, Kremsmunster, Austria). Subsequently, cells were incubated with 10 µм Fluo‐3/AM for 1 h. Subsequently, the CAP patch was treated for 5‐ or 10‐min. The fluorescence intensity was detected at an excitation wavelength of 485 nm and an emission wavelength of 520 nm immediately. Intracellular calcium levels were compared to those of untreated cells, which were set at a value of one relative fluorescent unit. To investigate to Ca^2+^ image in keratinocytes, cells (1 × 10^3^ cells per well) were seeded in ibidi µ‐Slide 8‐well high glass‐bottom plates: # 1.5H Schott glass. After 24 h, cells were treated with 10 µм Fluo‐3/AM (Invitrogen) for 1 h in 37 °C. Subsequently, CAP patch treatment was performed for 5 or 10 min. Next, the cells were incubated with DAPI for 2 min. Images were acquired using a Zeiss confocal laser scanning microscope (Zeiss microsystems, Germany) at 400× magnification.

### Ca^2+^ Gradient Imaging in Skin

Hanks’ Balanced Salt solution (HBSS) was mixed (1:1) with a stock solution (2 mm stock in dimethylsulfoxide) of Fluo‐3/AM (Invitrogen) before adding 1% HBSS buffer. Deparaffinization and rehydrate tissue sections were fixed using 4% paraformaldehyde. Next, the sections were stained at 4 °C for 3 h in the dark with 10 µм Fluo‐3/AM (Invitrogen) mixed with 1% BSA in HBSS. After staining, the sections were washed with PBS. The fluorescence signal was observed using a Zeiss confocal laser scanning microscope at an excitation wavelength of 488 nm.

### Immunocytochemistry for Tight Junctions in Keratinocytes

HaCaT (2 × 10^4^ cells per well) was cultured in Lab‐Tek chamber slides (Thermo Fisher). Cells were treated with a CAP patch for 5 or 10 min and then treated with TNF‐*α* (20 ng mL^−1^) and IFN‐*γ* (20 ng mL^−1^) for 18 h. Slides were washed three times with cold PBS. Subsequently, cells were treated with 4% paraformaldehyde for fixation (10 min) and were then permeabilized with 0.1% Triton‐X 100 for 15 min at 4 °C. After the slides were blocked with 10% (w v^−1^) BSA in PBS for 1 h at 25 °C, slides were incubated with anti‐occludin and anti‐claudin‐1 antibodies for 24 h at 4 °C. After washing with cold PBS, the slides were further incubated with Alexa Fluor 594‐conjugated goat anti‐rabbit IgG (Invitrogen) and Alexa Fluor 488‐conjugated goat anti‐mouse IgG (Invitrogen) for 1 h at 4 °C. The slide was washed with cold PBS and stained with DAPI for 2 min. Subsequently, the slides were coverslipped, and the fluorescence signal was visualized using a Zeiss confocal laser scanning microscope at 630× magnification.

### Patch Clamp

HaCaT was placed on glass coverslips coated with poly‐D‐lysine and grown in DMEM media (10% FBS) at 37 °C with 5% CO_2_ for 4 h before experiments. Whole‐cell voltage‐clamp recordings were performed at 25 °C to measure inward currents with Axopatch‐200B amplifier and 1440A Digidata (Axon Instruments). The patch pipettes were pulled from borosilicate capillaries. When filled with the pipette solution, the resistance of the pipettes was 5–6 MΩ. The recording chamber (300 µL) was continuously superfused (3–4 mL min^−1^). Series resistance was compensated for >80%) and leak subtraction was performed. Data were low‐pass‐filtered at 2 kHz and sampled at 10 kHz. The pClamp10 (Axon Instruments) software was used during experiments and analysis. For inward current recording, the pipetted solution containing 126 K‐gluconate, 10 mm NaCl, 1 mm MgCl_2_, 10 mm EGTA, 2 mm NaATP, and 0.1 mm NaGTP was adjusted to pH 7.4 with KOH. The extracellular solution composed of 140 mm NaCl, 5 mm KCl, 1 mm MgCl_2_, 2 mm CaCl_2_, 10 mm HEPES, and 10 mm glucose was adjusted to pH 7.4 with NaOH. A voltage clamp was performed at a holding potential of −70 mV.

### Statistical Analysis

Statistical analyses were performed using Prism 6 software (GraphPad Software, San Diego, CA, USA). Analysis was performed using a one‐way analysis of variances (nonparametric); Dunnett's multiple comparisons test. Statistically significant differences were defined as *p <* 0.05 and indicated by * *p <* 0.05, ** *p <* 0.01, *** *p <* 0.005, **** *p <* 0.0001, and # *p <* 0.05 compared with 5 min CAP patch.

## Conflict of Interest

The authors declare no conflict of interest.

## Author Contributions

Wrote original draft: N.K. and S.L. Performed the experiments: N.K., J.K., and Y.‐A. C. Fabricated the cold atmospheric patch: S.L. Performed the experimental methodology: S. L., J.P., and C‐K.P. Helped in supervising the experiment and writing‐review and editing: D.K. and S‐H.K.

## Supporting information

Supporting Information

## Data Availability

Research data are not shared.
