# Supplementary material for: Portable Cold Atmospheric Plasma Patch‐Mediated Skin Anti‐Inflammatory Therapy
Source: Adv Sci (Weinh). 2022 Sep 30;9(34):2202800. doi: 10.1002/advs.202202800 (PMC9731685; doi:10.1002/advs.202202800)
Supplement: Supplementary file 1 — Supporting Information [file ADVS-9-2202800-s001.pdf]

ADVANCED SCIENCE
Open Access

Supporting Information

for *Adv. Sci.*, DOI 10.1002/advs.202202800

Portable Cold Atmospheric Plasma Patch-Mediated Skin Anti-Inflammatory Therapy

Namkyung Kim, Seunghun Lee, Soyoung Lee, Jinjoo Kang, Young-Ae Choi, Jeongsu Park, Chul-Kyu Park, Dongwoo Khang and Sang-Hyun Kim**

Supplementary Figure Legends

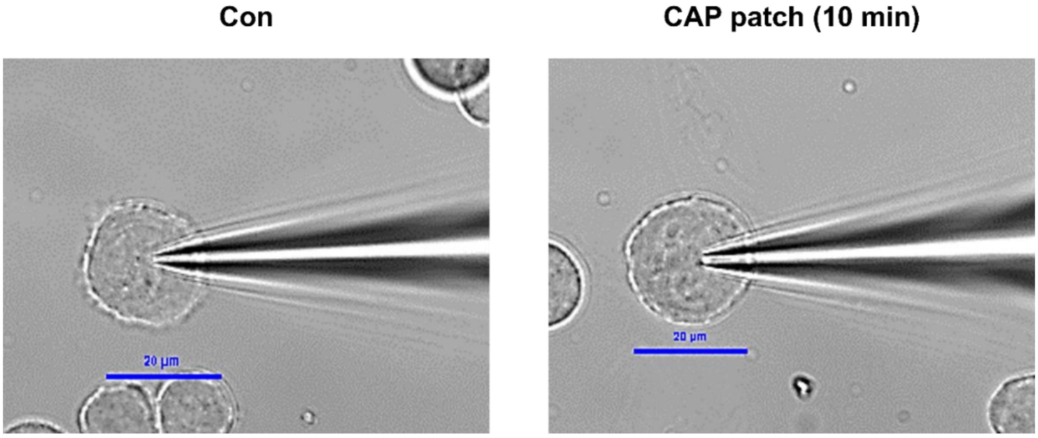

Figure S1. The image in patch-clamp after CAP patch (10 min) treatment. Visualization of cell morphology during patch-clamp recording on the left. Scale bar, 20 μm.

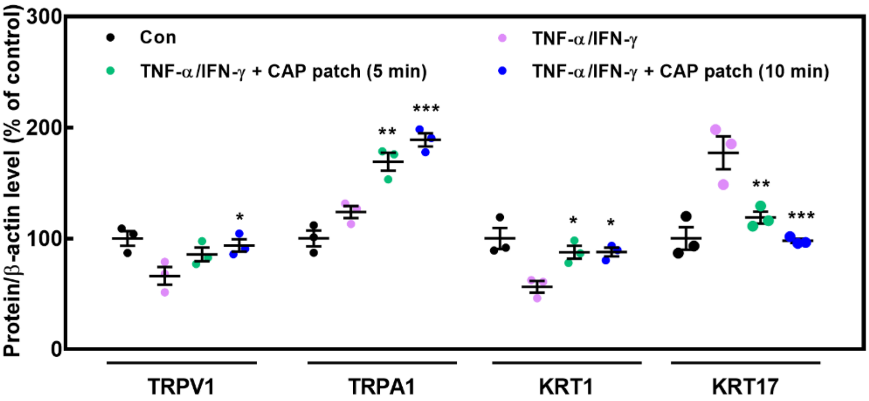

Figure S2. The CAP patch recovered the keratin and calcium channel proteins in keratinocytes. The band of β-actin was used as a loading control. The intensity of each band was quantified using Image J program. The graph represents the means ± SEM of three independent experiments.

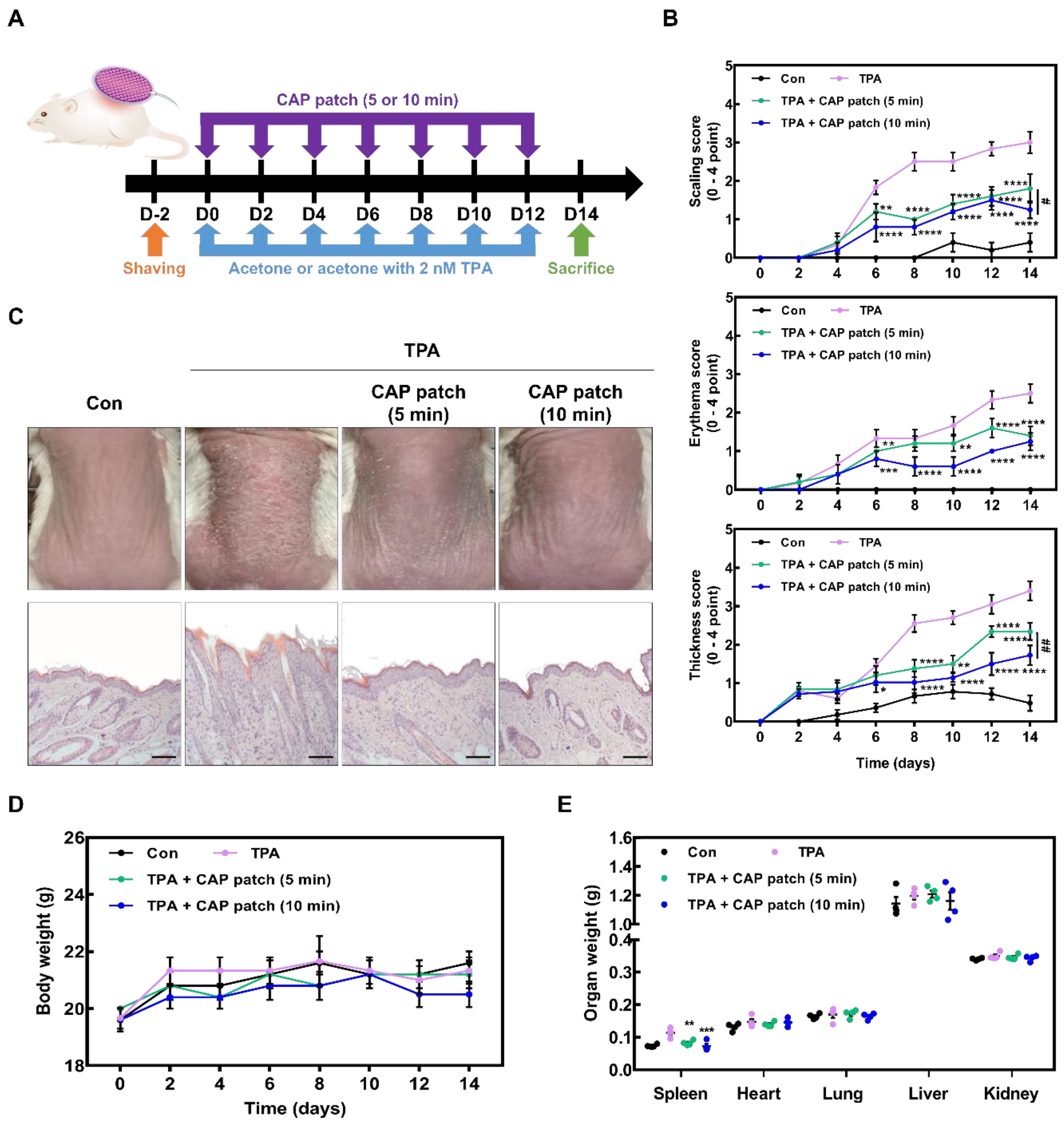

Figure S3. The CAP patch reduced psoriatic characteristics of 12-*O*-tetradecanoylphorbol-13-acetate (TPA)-induced psoriasis model. (A) The experimental scheme of CAP patch effect in TPA model. (B) PASI score of mice. The skin thickness was checked every 48 h using a dial thickness gauge. To PASI score, scaling, erythema, and thickness were measured from 0 to 4. (C) Phenotypical observation of mice skin and mice skin tissues were stained with H&E. (D, E) Organ weight and bodyweight of mice. Data are presented as the mean ± SEM ($n = 5$). * $p < 0.05$ compared with the TPA-induced group only. # $p < 0.05$ compared with TPA plus CAP patch (5 min).

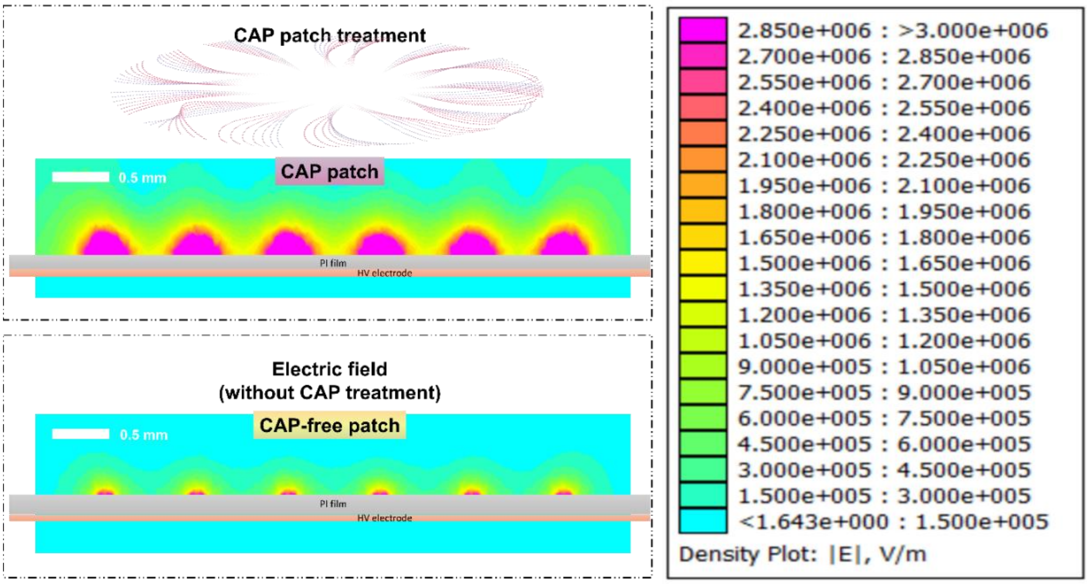

Figure S4. The distribution of electric field on plasma patch. (A) CAP patch has a 125 μm polyimide film. The thickness was suitable to generate surface air plasma by 2.4 kV peak voltage. (B) CAP patch has a 250 μm polyimide film. The thickness was very thick to generate surface air plasma by 2.4 kV peak voltage. Also, the pink region (|E| > 30 kV/cm) where plasma can be generated is too narrow to interact with skin contact.

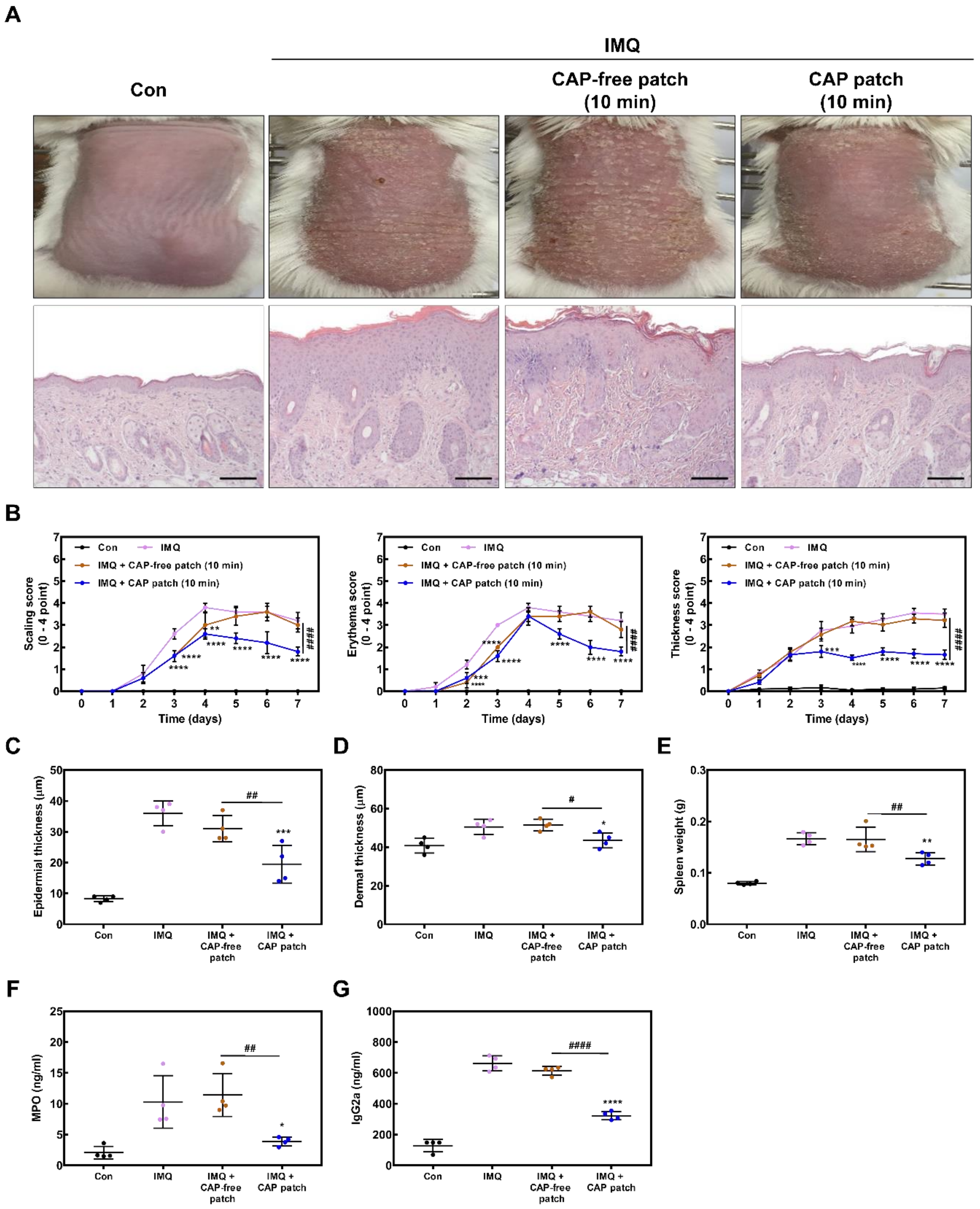

Figure S5. The electric field patch did not alleviate the psoriatic symptoms. (A) Phenotypic observations and H&E stain after CAP patch or electric field patch treatment. (B) PASI score was measured for psoriasis severity assessment after CAP patch or electric field patch treatment. (C, D) Epidermal and dermal thickness with the stage micrometer (200× magnification). (E) Spleen weight of mice after IMQ and CAP patch or electric field patch

treatment for 7 consecutive days. (F, G) After sacrificing, whole blood was collected. IgG2a and MPO were measured in serum through sandwich ELISA. Data are presented as the mean ± SEM (n = 5). * p < 0.05 compared with the IMQ-induced group only. # p < 0.05 compared with IMQ plus CAP-free patch (10 min).

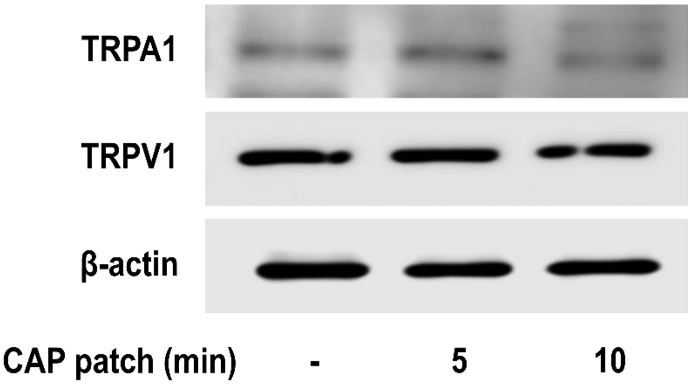

Figure S6. Calcium channels of normal skin by CAP patch. After 7 days of CAP patch treatment on normal mice skin, TRPA1 and TRPV1 were detected by Western blot.

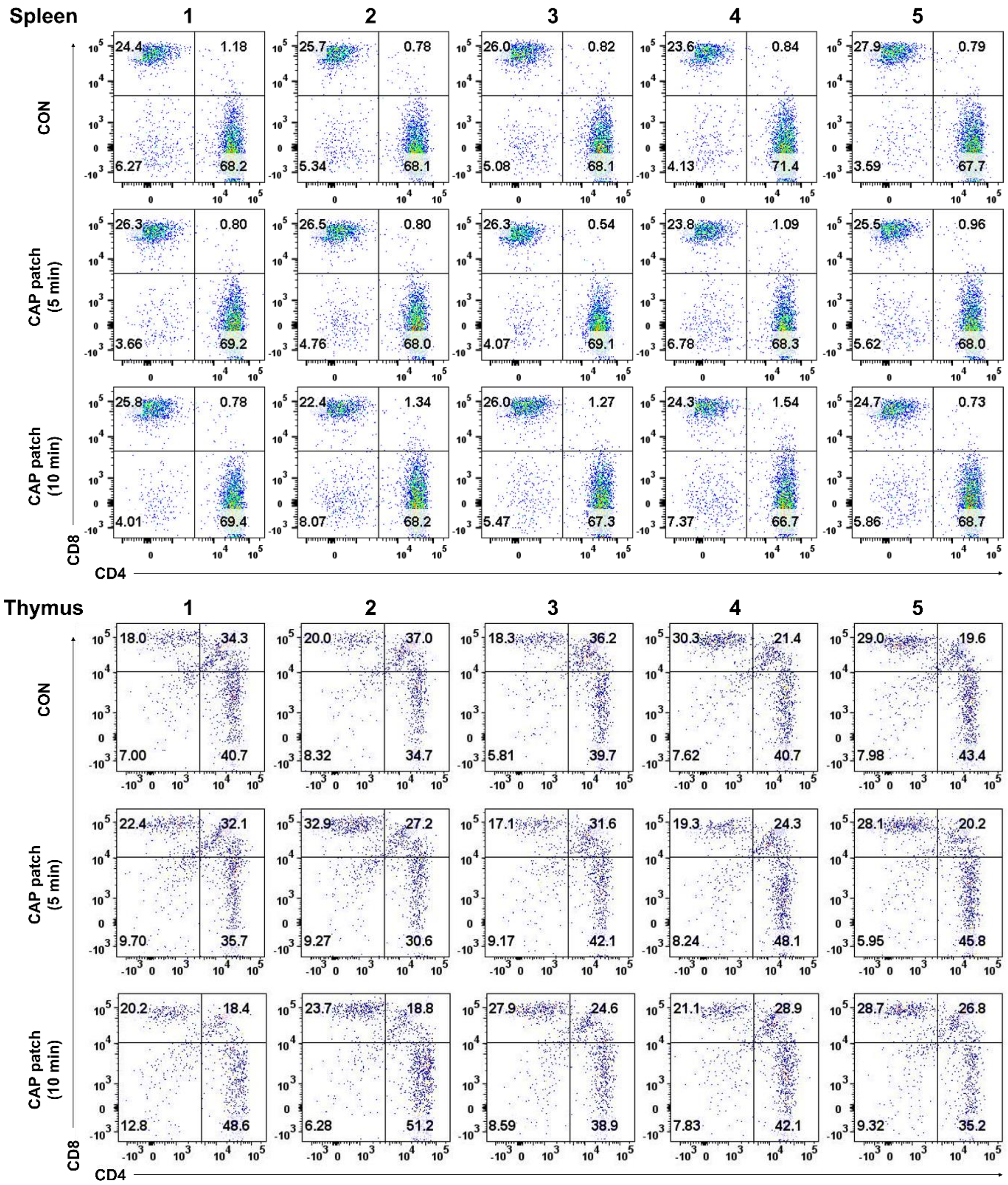

Figure S7. Flow cytometry analysis after CAP patch treatment to immunotoxicity evaluation. After 7 days of consecutive treatment with the CAP patch on normal mouse skin, the spleen and thymus were extracted and analyzed by flow cytometry.

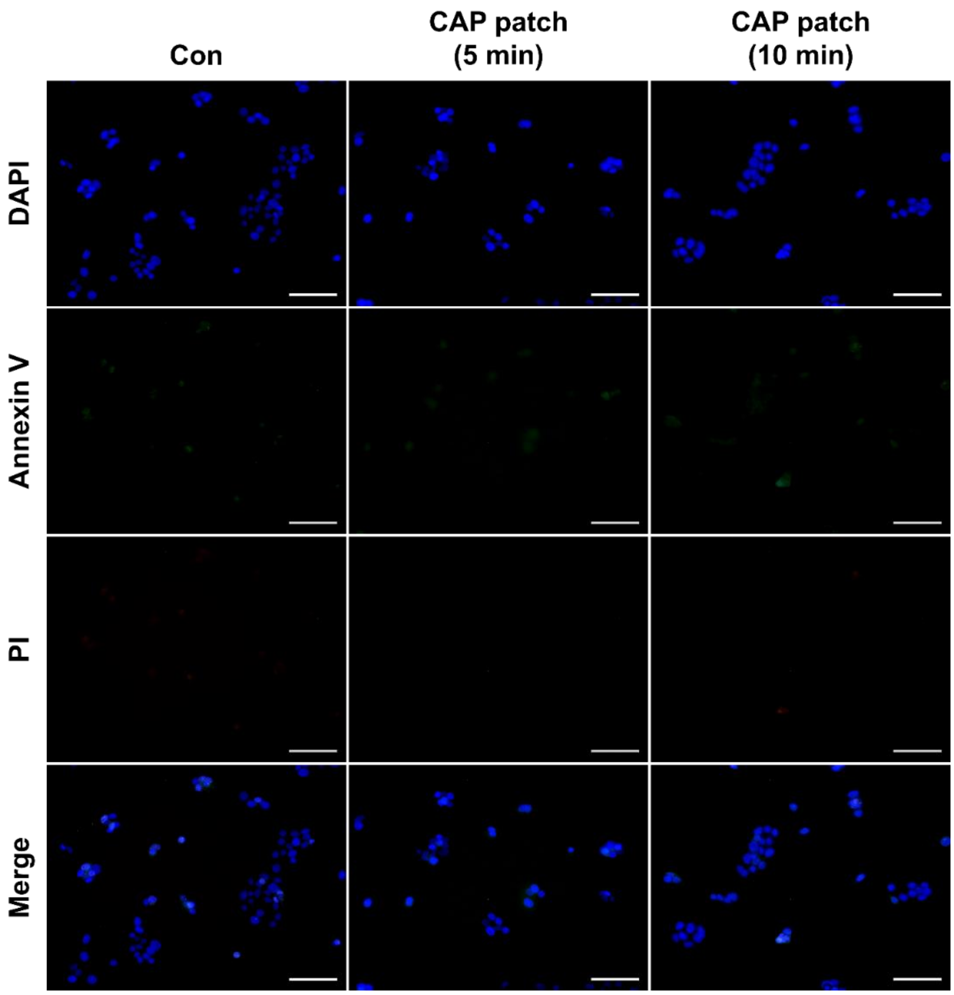

Figure S8. Cell death analysis by CAP patch treatment. The cell death of keratinocytes was assessed using the cell death assay. Keratinocytes with CAP patch (5 or 10 min) were cultured with the cells for 24 h, and the resultant apoptotic cells were stained with Annexin V and propidium iodide kit. The image of apoptotic or necrotic cells was obtained per five high-power fields. Blue = nuclei; Green = apoptotic cells; Red = necrotic cells. Scale bar = 100 μm, magnification 200×.

Table S1. Time weighted average (TWA) values according to the operating conditions of the CAP patch.

Frequency (Hz)	Peak voltage (kV)	O_3 (ppmv)	TWA
60	2.0	0.071	0.001
60	2.4	0.154	0.003
60	2.8	0.405	0.008
1000	2.0	9.5	0.198
1000	2.4	49.6	1.033
1000	2.8	86.8	1.808

Table S2. Specific primers and conditions used for experiments.

Primer / GenBank accession number	Sequence (5'-> 3')	PCR condition stage
***In vivo* (Mouse)**		
IL-17A NM_010552.3	F: TTT AAC TCC CTT GGC GCA AAA	**Holding – 1 cycle** 95 °C, 5 min **Cycling – 40 cycles** 95 °C, 10 sec 58 or 60 °C, 30 sec
	R: CTT TCC CTC CGC ATT GAC AC	
IL-36 NM_027163.4	F: GCT GTG TTG AGA TGG AGG GC	
	R: GAC AGA AGT GGA GCC CTC TA	
IL-1β NM_008361.4	F: ATA ACC TGC TGG TGT GTG AC	
	R: AGG TGC TGA TGT ACC AGT TG	
IL-6 NM_031168.2	F: CCA GTT GCC TTC TTG GGA C	
	R: GGT CTG TTG GGA GTG GTA TC	
CXCL1 NM_008176.3	F: TGT GGG AGG CTG TGT TTG TA	
	R: ACG AGA CCA GGA GAA ACA GG	
GAPDH NM 001289726.1	F: GCA CAG TCA AGG CCG AGA AT	
	R: CGG TTC TCC ATG GTG GTG AA	
***In vitro* (Human)**		
**Primer / GenBank accession number**	**Sequence (5'-> 3')**	**PCR condition stage**
IL-1β NM_000576.3	F: GCT GAT GGC CCT AAA CAG ATG AA	**Holding – 1 cycle** 95 °C, 3 min **Cycling – 40 cycles** 95 °C, 10 sec 58 or 60 °C, 20 sec
	R: TGA AGC CCT TGC TGT AGT GGT G	
CCL22 NM_002990.5	F: AGG ACA GAG CAT GGATCG CCT ACA	
	R: TAA TGG CAG GGA GGT AGG GCT CCT	
IL-6 NM_001371096.1	F: AAA GAG GCA CTG GCA GAA AA	
	R: ATC TGA GGT GCC CAT GCT AC	
CCL17 NM_002987.3	F: GTT CGG ACC CCA ACA ACA AG	
	R: TGG CTC CAG TTC AGA CAA GG	
IL-8 NM_000584.4	F: GGT GCA GTT TTG CCA AGG AG	
	R: TGC TTG AAG TTT CAC TGG CAT C	
GAPDH NM_002046.7	F: CGA CCA CTT TGT CAA GCT CA	
	R: AGG GGA GAT TCA GTG TGG TG	

IL, Interleukin; CCL, Chemokine (C-C motif) ligand; CXCL, Chemokine (C-X-C motif); GAPDH, Glyceraldehyde-3-phosphate dehydrogenase.

Table S3. Antibodies used for Western blot.

Target protein	Supplier	Cat#	Host	Dilution
TRPV1	Novus Bio, Littleton, CO	NBP1-97417	Rabbit	1:1000
TRPA1	Novus Bio	NB110-40763	Rabbit	1:1000
KRT1	Santa Cruz Bio, Dallas, TX	SC65999	Mouse	1:1000
KRT14	Abcam, Cambridge, UK	Ab7800	Mouse	1:1000
KRT17	Santa Cruz Bio	SC393002	Mouse	1:1000
β-actin	Invitrogen, Waltham, MA	MA5-15739	Mouse	1:2000

TRPV, Transient receptor potential cation channel subfamily V member; TRPA, Transient receptor potential cation channel subfamily A member; KRT, Cytokeratin
